# Implications for Dietary Guideline Policy of a Cultural Adaptation of the US Dietary Guidelines for Women of Mexican Descent: A Pilot Study

**DOI:** 10.3390/nu17223578

**Published:** 2025-11-15

**Authors:** Norma Garfias-Avila, Ching-Yun Wang, Johanna W. Lampe, Jason A. Mendoza, Jean De Dieu Tapsoba, Norma J. Alcalá, Lisa Levy, Marian L. Neuhouser

**Affiliations:** 1 Food Systems Nutrition and Health Program, University of Washington, Seattle, WA 98195, USA; 2 Division of Public Health Sciences, Fred Hutchinson Cancer Center, Seattle, WA 98109, USA; cywang@fredhutch.org (C.-Y.W.); jlampe@fredhutch.org (J.W.L.); jay.mendoza@seattlechildrens.org (J.A.M.); llevy@fredhutch.org (L.L.); mneuhous@fredhutch.org (M.L.N.); 3 Department of Epidemiology, University of Washington, Seattle, WA 98195, USA; 4 Department of Pediatrics, University of Washington, Seattle, WA 98195, USA; 5 Seattle Children’s Research Institute, Seattle, WA 98101, USA; 6 Vaccine and Infectious Disease Division, Fred Hutchinson Cancer Center, Seattle, WA 98109, USA; jtapsoba@fredhutch.org; 7 Global Oncology, Fred Hutchinson Cancer Center, Seattle, WA 98109, USA; nmarisca@fredhutch.org

**Keywords:** traditional Mexican diet, randomized controlled trial, lifestyle intervention, biomarkers, nutrition policy

## Abstract

**Background/Objectives**: This study aims to evaluate whether the Dietary Guidelines for Americans (DGA) are effective for maintaining a healthy diet among Mexican-descent populations in the US or if a more culturally tailored policy approach is warranted. **Methods**: As a first outcome, 20 healthy women of Mexican descent from the Seattle area participated in a pilot randomized controlled trial. They were randomly assigned (10 participants each) to either a group receiving instruction on the standard 2015 DGA or a group receiving an adaptation of the DGA focused on traditional Mexican cuisine and culture. In this 12-week study (with follow-ups at 3 and 6 months), participants’ acceptability of the cultural adaptation of the DGA was compared with that of the standard DGA with end-of-study surveys. Ten blood-based metabolic biomarkers were assessed at baseline and 3 months. Dietary changes at 3 months were assessed with a Food Frequency Questionnaire (FFQ) that was translated into Spanish but not culturally adapted. The secondary outcome was dietary change at 6 months. **Results**: The primary findings at 3 months showed that serum free fatty acids were reduced for the standard DGA arm. Carbohydrate consumption was reduced in the standard DGA arm only. The end-of-study survey results suggested that both interventions were well received by participants. **Conclusions**: The preliminary findings from this small sample size suggest that depending on a person’s priorities, either intervention could be offered, with each arm showing slightly different dietary and biomarker outcomes.

## 1. Introduction

The largest population of foreign-born individuals in the US is of Mexican descent [1]. They experience several health disparities, which might render them an important focus of public health nutrition efforts. For example, they have a diabetes prevalence rate of 14.4%, whereas non-Hispanic whites have a diabetes prevalence rate of 7.5% [2]. Additionally, Mexicans living in the US have a much higher rate of obesity than non-Hispanic whites [1,3]. Hispanics (including Mexicans) have lower rates of health insurance, with coverage for people of Mexican descent at about 47.9%, compared with 74.7% for non-Hispanic whites [4,5]. Notably, recent Mexican immigrants often experience a decline in health in the years shortly after they migrate to the US from Mexico [6].

Acculturation plays an important role in the health of this population. Higher acculturation and lower socioeconomic status are associated with an increase in health deterioration in Mexicans living in the US [7]. Higher acculturation is associated with the consumption of less traditional Mexican diets and higher intake of processed foods [8]. Shifts in dietary patterns can be concerning because of the specific foods and nutrients that are being substituted, such as polyunsaturated fats and whole grains [9]. The micronutrient composition of the diet might also change significantly when living in the US, as Mexico-born children and adults have different—and often higher—serum concentrations of carotenoids and other antioxidants when compared with US-born Mexicans [10,11].

To complicate matters, dietary acculturation changes can also create micronutrient deficiencies. A study that used NHANES data found that over 25% of Mexican-descent women in the US are not meeting their estimated average requirement (EAR) for calcium, folate, magnesium, vitamin A, vitamin C, and vitamin D [12]. While some micronutrient deficiencies might be present even for Mexicans living in their home country, some deficiencies might be uniquely relevant in US-based populations [10,13]. Namely, in Mexican-descent children born in the US, serum concentrations were lower with respect to vitamin A and E when compared with their Mexican-born peers [10]. Therefore, current dietary guidelines might not be particularly effective for targeting the needs of ethnic and racial communities within the US [14].

This leads us to the importance of studying the food and food culture of Mexican-descent populations. The value placed on traditional Mexican food and culture plays a role in food choice. In particular, for women of Mexican descent, the preparation of culturally relevant foods is a common practice, and it embodies the expression of cultural traditions and helps pass on traditions to the next generation [15]. Parents of Mexican descent (mainly women) recognized that preparing traditional Mexican meals and sharing family mealtime enhances the enjoyment of food [16]. A study found that interventions targeting Mexican mothers could have an impact on the dietary patterns of their children [17].

Previous clinical-trial-based health behavior interventions aimed at improving dietary outcomes in the Mexican population have used promotoras (lay health workers) and focused on family-based and community-based interventions [18,19,20,21]. In a study with promotoras, electronically delivered information (newsletters) and periodic visits from the promotoras improved health-promoting family behaviors, namely, parenting strategies for eating and physical activity at the 2-year follow-up [22]. Other pilot studies that targeted this population found that culturally appropriate care is important for participants [23,24,25]. Additionally, some studies observed positive effects, such as increased attendance at nutrition information sessions, weight loss, and a reduction in insulin resistance scores [23,25,26]. These studies have used a variety of tools, including a strong focus on women, traditional Mexican food and practices, group activities, and partnering with community groups [23,24,25,26]. Thus, policies focusing on the traditional Mexican diet, and specifically on women of Mexican descent, could help increase the positive effects of dietary recommendations [17,18,19].

Given the specific characteristics of the Mexican population in the US, health policies that focus on nutrition need to be tailored and should take the social determinants of health, culturally relevant meals, and other food-related behaviors into consideration [15,16,26,27]. This might suggest that nutrition policies need cultural adaptations to be fully effective for people of Mexican descent living in the US. Moreover, the Dietary Guidelines for Americans (DGA) are not actively considering cultural practices despite being used to inform many federal programs [14]. One method of investigating whether nutrition policies need to be culturally tailored is to test the DGA vs. culturally modified guidelines while examining short-term outcomes such as biomarkers and dietary change. We hypothesized that first- and second-generation Mexican immigrant women would be more responsive to a culturally tailored adaptation of the DGA. Consequently, the objective of this study, the COMIDAS at home, was to understand whether a culturally tailored DGA with potential applications for policy guidance is more suited for a female Mexican-descent population living in the US compared with the standard DGA translated into Spanish without cultural adaptation.

## 2. Materials and Methods

This study was built on a parent study called Comparing Original Mexican Diets and Standard US diets (COMIDAS), which was a randomized crossover feeding trial that aimed to measure and compare the metabolic responses of 53 women of Mexican descent after consuming a traditional Mexican diet and a standard American diet. The methods and primary results are described in another publication [26]. The COMIDAS-at-home was a home-based pilot sub-study that used the same traditional Mexican diet from the parent COMIDAS trial, but instead of being a randomized crossover controlled feeding trial, it consisted of a randomized trial of behavioral intervention trial comparing eating patterns. Participants received instruction on which foods to consume and how to prepare them. The participants purchased and prepared all foods in their own homes following this study’s guidance. The COMIDAS-at-home intervention consisted of two parallel arms, where one arm used study staff to provide instruction on the 2015 DGA (standard DGA arm), which included Spanish translations provided by the United States Department of Agriculture (USDA), and the other arm consisted of staff-provided instruction on an adaptation of the DGA, which included traditional Mexican foods and cultural aspects of the diet (Mexican adaptation arm). This mixed-method study collected blood samples, body measurements, and qualitative surveys. The Fred Hutchinson Cancer Center’s Institutional Review Board approved all study protocols and procedures, and all participants signed written informed consent. All procedures used in this study adhered to the principles of the Declaration of Helsinki. The clinicaltrials.gov identifier is NCT01369173.

### 2.1. Participants

The study participants were first- and second-generation women of Mexican descent, aged 18–50 years, and in good health. Eligibility criteria included having one or two parents born in Mexico or being born in Mexico, residing in the Seattle area for the 6 months following the start of the trial, and willingness to follow study guidelines. Exclusion criteria included having food intolerances or allergies that would preclude following this study’s dietary recommendations, being pregnant or breastfeeding, being outside the eligible age range, or unwillingness to follow the study’s guidelines.

Twenty-four women were recruited from the parent COMIDAS study via email, and fliers were also distributed at a community clinic. Community-targeted social media ads were posted. 20 consented to participate. All 20 were screened and found to be eligible, after which they consented, enrolled, and were randomized to either the standard DGA arm or the Mexican adaptation arm. All study materials were available in English and Spanish. The study was conducted from 2016 to 2017. Enrolled participants completed several at-home pre-study activities, including a baseline questionnaire collecting demographic characteristics and other standardized information such as occupation, language used at home, marital status, and education; a baseline Food Frequency Questionnaire (FFQ); and the consent form.

The consent form was provided ahead of time to allow participants sufficient time to review it, but signatures were only collected in person after staff reviewed the consent form with participants and all questions were answered. Staff reviewed the completed questionnaires for the sake of completeness. An FFQ was collected at the beginning and end of this study, and this FFQ was only validated for the general population. All pre-study activities were designed to be completed in a single day, and participants had an approximate window of 2 weeks to complete these activities before the first clinic visit. The total intervention time was 3 months, and the total follow-up time was an additional 3 months for a total of 6 months.

### 2.2. Data Collection

Participants attended two in-person clinic visits. At visit one, participants submitted the completed questionnaires and consent form, and their height and weight were measured by staff using a standardized protocol. Next, they were randomized to receive study-provided instruction (behavioral intervention) on either the 2015 DGA or the Mexican adaptation for the next three months. The Mexican adaptation was based on a menu designed for the original study, with a focus on pre-Hispanic foods and cultural eating practices common in Mexican culture [26]. Ten participants were randomly assigned to each arm. Dietary intake data were collected using the Fred Hutchinson Cancer Center’s General Nutrition Assessment FFQ. This FFQ has 140-line items and 19 adjustment questions, and the reference period is “in the past month.” The FFQs were processed at the Fred Hutchinson Cancer Center’s Nutrition Assessment Shared Resource, which utilizes the Nutrition Data System for Research (NDSR) food and nutrient database software (version 2020, Nutrition Coordinating Center, University of Minnesota). NDSR uses responses from the FFQ to estimate daily intake per person for 140 nutrients and other compounds, including phytochemicals. Blood samples were collected on the first day of this study at visit one after a 12 h overnight fast and again after 3 months of study activities at visit two. Samples were processed and stored at −80 °C until laboratory analysis.

At visit one, bilingual staff provided individual instruction on the standard DGA or the Mexican adaptation, in accordance with each participant’s randomization arm. Study notebooks were given to all participants, which included study-provided recipes, sample menus, and other dietary tips that followed the dietary pattern. After visit one, the remainder of the intervention was delivered remotely through structured phone calls and text messages. Participants were free-living and were instructed to prepare their food using study materials. After 12 weeks, the participants were asked to complete another FFQ and blood draw; have their height and weight measured; and complete a qualitative survey about ease of use, satisfaction, barriers and facilitators, and overall experience. A final FFQ was completed 6 months later via mail.

### 2.3. Quantitative Analysis Measures and Analysis

Blood samples were assayed for glucose, insulin, lipid panel, and high-sensitivity *C*-reactive protein (hsCRP) at the Northwest Lipid Research Laboratories. The lipid panel consisted of total cholesterol, triglycerides, high-density lipoprotein (HDL), low-density lipoprotein (LDL), and very-low-density lipoprotein (VLDL), and it was assayed using standard enzymatic assays via an automated Roche analyzer. Glucose was analyzed using a clinical chemistry autoanalyzer. Insulin was quantified via a 48-h PEG-accelerated, double-antibody radioimmunoassay. The Homeostatic Model Assessment (HOMA) for insulin was computed through the ratio of insulin and glucose levels: Insulin (µU/mL) × Glucose (mg/dL)/405. hsCRP was assayed using a Roche Cobas Mira chemistry analyzer. Finally, free fatty acids (FFAs) were assayed at the Fred Hutch Public Health Sciences Biomarker Laboratory via the enzymatic colorimetric method using kits from Wako (Richmond, VA, USA).

The coefficient of variation (CV) for insulin was 8.8% using study-embedded blinded duplicate quality control samples. The remaining CVs were 0.9%, 0.9%, 1.9%, 2%, 0.8%, 3.1%, 1.4%, and 1.2% for cholesterol, HDL cholesterol, LDL cholesterol, VLDL cholesterol, glucose, hsCRP, triglycerides, and FFAs, respectively. Participants whose fasting blood values exceeded 200 md/dL for total cholesterol, 130 mg/dL for LDL-cholesterol, or 125 mg/dL for glucose were provided with an IRB-approved letter containing the measured value and were urged to take the letter to a healthcare provider. The letter included the names and addresses of local clinics with sliding scale fees. There were four participants with LDL cholesterol > 125 mg/dL and two participants with glucose > 125 mg/dL.

Plots were created and used to examine the distribution of the blood biomarker data. Next, Kolmogorov–Smirnov, Shapiro–Wilk, Cramer-Von Mises, and Anderson–Darling tests were calculated to test the normality of data distributions. Most data were determined to be non-normally distributed and were transformed using the ln function. Data from dietary questionnaires were determined to be normally distributed, but they were transformed for standardization, as is commonly carried out for dietary intake data. Moreover, geometric means were used in the analysis.

To compare both arms at baseline, *t*-tests and χ^2^ tests were performed. Two-sided *t*-tests were used to compare continuous demographic variables and geometric dietary intake means. An χ^2^ test was used for categorical demographic variables. Standard deviations were also calculated for all baseline characteristics, in addition to percentages for categorical values.

For comparisons of the effects of each arm on biomarkers and diet, generalized estimating equations (GEEs) were computed, and *p* values of ≤0.05 were considered statistically significant. GEE was adjusted for age and BMI. Analyses were conducted using R statistical software version 4.0.2 (R Foundation for Statistical Computing, Vienna, Austria). Primary analysis was used to examine changes after 3 months, and secondary analysis was applied to investigate changes after 6 months. The intervention effects of the standard DGA vs. Mexican adaptation recommendations on biomarkers and diet were evaluated by contrasting the measures at baseline and after 3 and 6 months, unadjusted and adjusted for age and BMI.

### 2.4. Qualitative Analysis: End-of-Study Survey

Out of the 10 questions in the end-of-study survey, 8 were qualitative and 2 were quantitative, and these were measured using a Likert scale. For the 2 quantitative questions that asked about ease of use and understanding, percentages were calculated. The 8 qualitative questions were designed to understand whether the intervention was well received and whether it was useful, as well as to obtain general participant feedback. An inductive iterative approach was used to create a codebook and analyze the responses to the 8 questions [28]. To create the codebook, all responses were read first, and codes were created afterwards. Later, themes were created, and codes were assigned to different themes. Additional subthemes and codes were later added after all data were examined. Although all responses were coded in the same manner, responses from different arms were color-coded to facilitate the analysis of possible differences between each arm. All interviews were manually coded by a single person using Microsoft Word version 2023. Codes were reviewed and approved by three other people in supervisory roles.

## 3. Results

**Figure 1:**
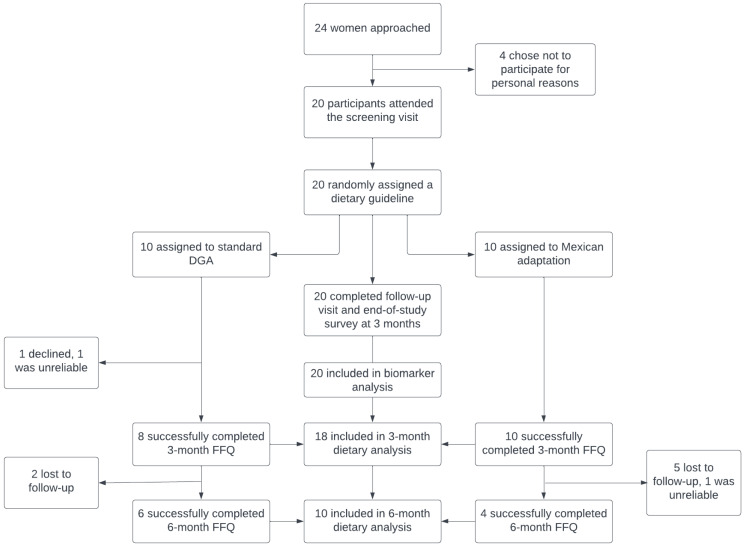
Consort diagram of study enrollment (created using Lucidchart 2025).

A total of 20 women signed the consent form and were enrolled in this study. Figure 1 shows the Consort diagram of this study’s enrollment, randomization, and completion. Table 1 describes the demographic characteristics and other baseline characteristics of the participants. All participants completed blood draws at baseline and after 3 months (12 weeks), as well as the end-of-study survey. A total of 19 women completed the second FFQ at 3 months, but only 11 women completed a third FFQ at 6 months, which limited the statistical power and population generalizability. Completed FFQs reporting less than 600 kcal/day were considered unreliable, which resulted in 18 and 10 FFQs included for the 3- and 6-month dietary analyses. The reasons for non-completion comprised loss to follow-up (*n* = 7) and refusal (*n* = 1). Participants’ body weight remained stable for both diet arms after 3 months, as evaluated via a GEE model adjusted for age and baseline weight (*p* = 0.85).

The mean differences between both arms were relatively similar (Table 2), and GEE models showed that the difference between pre-intervention and post-intervention serum FFAs was the only biomarker with a statistically significant *p*-value before and after adjusting for age and BMI. FFAs were reduced by 26% in the standard DGA arm and increased by 9.8% in the Mexican adaptation arm, resulting in a 35.8% difference between the arms. Total serum cholesterol was not statistically significant after adjusting for age and BMI.

Intervention effects on dietary components varied according to follow-up time and components (Table 3). Between baseline and the 3-month follow-up (*n* = 18), carbohydrate consumption differences in g/day between groups amounted to 29%, with the standard DGA group exhibiting a 31% reduction. The share of protein intake was slightly diminished for the Mexican adaptation arm after 3 months. While not statistically significant, vegetable intake appears to have increased in the Mexican adaptation arm.

As for the secondary analysis with 10 participants at 6 months (see Figure 1 for study enrollment and Table 4), the total energy (kcal/day), carbohydrate (g/day), total fat (g/day), saturated fat (g/day), and protein (g/day) differences between each group were statistically significant. The Mexican adaptation group had a marked decrease in energy intake, which also reduced the intake of other nutrients.

### End-of-Study Survey Results

Questions 1 and 2 had quantitative response options using a Likert-type scale and asked how easy or difficult it was to understand and use the study-provided eating guides, respectively. There was a slight preference towards the standard DGA arm: 70% reported it was very easy to understand the DGA eating guide vs. only 40% in the Mexican adaptation arm (*p* = 0.007). For question 2, 90% of standard DGA participants reported the eating guide as very easy or easy to understand vs. 60% of the Mexican adaptation participants (*p* = 0.16). The end-of-study questions are outlined in Table 5.

For the qualitative component, a total of 38 unique codes were created and coded. Four total themes were identified: factors contributing to adherence to the intervention, factors negatively affecting adherence to the intervention, perceptions of specific dietary components, and information or practices learned from the intervention and suggestions.

The first theme, factors contributing to the adherence of the intervention (Table 6), included positive comments about the intervention, including components, usefulness, feasibility, importance, and ease of adaptation. Ease of use was the code with the greatest number of comments. Most intervention components, such as calls, texts, and in-person guidance, received an overwhelming majority of positive comments. The only notable difference between the arms was the code variety, for which no comments were received from the Mexican adaptation arm.

The second theme was factors negatively affecting adherence to the intervention (Table 7), and this theme included comments on aspects of the intervention that were difficult to follow; intrinsic factors such as lack of time; and extrinsic factors that hindered adherence, such as family pressure. Overall, negative comments or reported barriers were significantly less common than positive ones. Most factors or barriers affecting adherence were not related to this study but rather related to lack of time, dislike for certain foods, pushback from their families, and others. Only one factor from this study was reported as having a negative impact on participants’ experience—the timeliness of study materials—which was reported in both arms. This code captured several participant comments indicating a preference for receiving the recipes earlier in the study.

The third theme, perceptions of specific dietary components (Table 8), included all mentions (positive, negative, or neutral) of distinct dietary components. The codes included fruits and vegetables, fat, sugar, sugar-sweetened beverages, junk food (in general), water, salt, meats, beans, flours, and other previously unclassified components. Codes with predominantly negative comments, or those indicating a desire to reduce consumption of a dietary component, were mostly observed for fat, sugar, sugar-sweetened beverages, junk food, salt, and flour. Comments with a more positive outlook, or those indicating a desire to increase consumption, were associated with the codes for fruits and vegetables, water, and beans.

Finally, the fourth and final theme was information or practices learned from the intervention and suggestions (Table 9). Codes included useful information learned, changes made during the intervention, desire for continuation of changes post-intervention, portion sizes, frequency, exercise, and suggestions. Suggestions included ideas such as keeping a food record, providing more information about diseases, timely delivery of materials, and creating guides for children and busy individuals.

## 4. Discussion

The COMIDAS-at-home pilot sub-study tested the extent to which US-policy-based dietary recommendations (standard DGA vs. Mexican adaptation) given to free-living women of Mexican descent could improve their dietary intake and health status. This study also aimed to determine whether the traditional Mexican diet was not only potentially more effective at improving health than the standard DGA but also more feasible, culturally appropriate, or easily adopted by participants. After 3 months, the standard DGA arm exhibited significant improvements with respect to serum FFAs. The standard arm reported baseline and follow-up geometric mean FFAs measures as 0.50 and 0.37, respectively, and for the Mexican adaptation arm, the values were 0.41 and 0.45. Some modest changes were also clinically relevant but not statistically significant with respect to the reduction in total cholesterol and nutrient intake for the standard DGA arm. Due to the small sample size and exploratory nature of this study, more research is needed to confirm whether these measures are indeed responsive to these types of interventions. Furthermore, the participants of the standard DGA arm exhibited a small decrease in serum triglycerides.

In the qualitative analysis, participants overwhelmingly reported positive comments with respect to both arms. However, the participants of the standard DGA arm reported slightly higher ease of understanding and use regarding their provided materials and guides. It is possible that participants were already familiar with some of the standard DGA materials, as they are publicly available documents. Furthermore, participants in the Mexican adaptation arm may have had their food choices limited by external factors, such as food availability. Additionally, since we did not provide any food, it is possible that participants in either arm were affected by food insecurity. Food insecurity is linked to factors such as immigration status [29]. Minority populations are more affected by food insecurity, and Mexican immigrants face particularly high food insecurity rates; they are less likely to participate in the SNAP (Supplemental Nutrition Assistance Program) [30]. This study does not evaluate experiences of interpersonal or structural racism, or their potential impact on diet quality or health outcomes.

The diet intervention induced changes in serum biomarkers in both arms. Overall, at 3 months, the decrease in total dietary energy intake, along with carbohydrate and fat intake (when compared with the Mexican adaptation arm), could account for the decrease in FFAs and possibly cholesterol in the standard DGA arm’s participants. Increased serum FFAs concentrations have been associated with insulin resistance and overall inflammation [31]. These results contrast with the results obtained in the original study, in which CRP and insulin improved after the feeding trial for the Mexican adaptation arm. However, in the parent study, the comparison group was not provided with a DGA-adapted diet but rather with a standard American diet based on NHANES data. Nevertheless, the functions and effects of FFAs on the body vary depending on their chain length and degree of saturation [32].

The small increase in vegetable intake in the Mexican adaptation arm could be particularly important, given that this population may be deficient in multiple micronutrients, as vegetables are a rich source of vitamins and minerals, including potassium, folate, carotenoids, vitamin E, zinc, magnesium, and phosphorus [33]. Protein intake monitoring appears to be vital for this population, particularly as the Mexican adaptation arm revealed decreased proportional protein consumption, and all participants obtained less than 20% of their total daily energy intake from protein. Despite this, it is worth noting that participants in the Mexican adaptation arm did not report a lack of time to prepare food. This could potentially be explained by their familiarity with the recommended foods. Participants of both arms reported having some degree of family pushback, highlighting the importance of understanding family dynamics for this population.

Given the large number of Mexican-descent individuals in the US, this pilot intervention observed that tailored nutrition recommendations with culturally relevant meals and other food-related behaviors could be useful and have policy implications. It may be important not only to translate consumer-facing nutrition policy documents into Spanish but also to conduct qualitative analyses to ensure that cultural aspects of food and eating are properly addressed and understood. Such attention to policy development could improve dietary intake and ensure the adequacy of both macronutrients and micronutrients. Participant feedback indicated overall feasibility in following and understanding the diet, as well as its cultural relevance. The availability of culturally adapted DGA could help address health disparities in some of these populations by making evidence-based nutrition recommendations more accessible and practical to implement within existing cultural food practices. This would thereby help reduce barriers to healthy eating that stem from cultural disconnection with respect to standard dietary guidelines. For Mexican populations struggling with micronutrient deficiency, the 3-month increase in vegetable consumption observed in the Mexican adaptation arm indicates that this option might be particularly valuable for them. Similarly, for individuals who value Mexican food, the Mexican adaptation might be especially useful when explaining nutrition requirements. Conversely, if patients or providers prefer to prioritize lipid profiles, the reduction in FFAs in the standard DGA arm might be more appropriate.

### Strengths and Limitations

Strengths of this study include the fact that culturally tailored dietary intervention trials recommending a traditional Mexican diet and delivered via text and phone have not been studied in the Mexican-descent population in the US. However, these approaches have been moderately accepted in other populations [34,35,36]. A novel contribution of this study was the mixed-method use of both quantitative and qualitative analyses. More strengths of this study include the incorporation of qualitative data, being the first of its kind to evaluate guideline recommendations among free-living individuals who choose and prepare their own meals, and the re-evaluation of dietary intake 3 months after the end of this study (6 months after the start of the intervention). Despite its small sample size, we were able to find differences in FFAs values between each arm. Behavioral intervention trials with free-living individuals are particularly helpful when trying to decide on policy, as their conditions more closely resemble the natural environments of populations. This can be particularly helpful when considering dietary recommendations and policy.

A limitation was that some participants reported not having recipes to prepare food and noted that providing this information at the beginning of the study would have helped them follow each guideline more effectively. Acculturation was not accounted for in our population. Future studies, as well as policy guidance, could incorporate the lessons learned from this study. It is important to note that only one person coded all survey results, which might render them more susceptible to bias. In addition, the end-of-study questionnaire on participant experiences was not formally validated.

Additional limitations include the location of this study and its small sample size. All participants were from the Seattle area, which might not reflect the eating habits and nutritional intake of individuals of Mexican descent in other parts of the US. Although several measures of dietary intake were significantly different at 6 months between each arm, data are likely unreliable not only because of the small number of participants and FFQs that were included in the analysis but also because the mean energy intake of the Mexican adaptation arm was ~983 kcal/d. The high attrition for the six-month FFQ resulted in a loss of statistical power, increasing the risk of a Type II error. Moreover, the low mean energy intake might point to a lack of understanding or underreporting by participants. For both the 3-month and 6-month diet data, it was only possible to obtain one FFQ for each dietary assessment, which further limits the reliability of dietary intake data, as one FFQ might not be sensitive enough to detect dietary changes. The FFQ used might not be sufficiently culturally adapted to capture all culturally relevant foods, which could potentially explain why the Mexican adaptation arm’s mean energy intake at 6 months was below 1000 kcal/d. A culturally adapted FFQ could be used to improve this, as the FFQ was translated into Spanish but not explicitly validated in a population of Mexican descent. A 24-h recall interview administered on at least two different days—or a multiple-day food diary—could further increase reliability in future studies. Finally, a limitation of this study was that we could not distinguish which types of FFAs changed in our population.

This pilot study only included 20 participants, but a follow-up study could address shortcomings. Lessons learned include increasing sample size, better dietary intake data collection, validation of the end-of-study questionnaire, and improved coding procedures. Further, attrition rates should be carefully monitored, and incentives to keep participants enrolled could help with retention. These changes could help determine not only whether our results are replicable but also whether a cultural adaptation of the DGA would result in improved biomarker profiles and diet. A larger, national study could therefore help future dietary guideline policies. Likewise, our study only examined a Mexican adaptation, but further studies could test other cultural adaptations for different immigrant populations in the US.

## 5. Conclusions

Behavioral intervention studies of this type with this population had not been done before. This study contributed to the understanding of how a traditional Mexican diet is perceived and valued by Mexican-descent women. It also contributed to the literature of behavioral trials, and the lessons learned can help further studies. Concerning the use of culturally adapted dietary guidelines, these preliminary results based on a small sample size suggest that both the standard DGA and the adapted one could be used in US populations of Mexican descent. Both could be used as a tool for dietary intervention and for general policy-based dietary guidance. While the results are preliminary, providers could recommend a dual offering to their clients and patients for nutrition education, tailored to their individual goals, preferences, or values.

## Figures and Tables

**Table 1:** Baseline demographic characteristics of 20 subjects.

	All	Standard DGA Arm	Mexican Adaptation Arm
Age (years) *	38.7 (8.6)	41.1 (8.0)	36.2 (8.9)
Weight (kg) *	73.0 (19.4)	77.5 (21.8)	68.6 (16.5)
BMI (kg/m^2^) *	29.9 (7.4)	31.6 (7.5)	28.3 (7.3)
Energy intake (kcal/d) *	1685 (628)	1742 (528)	1627 (739)
BMI (kg/m^2^) ^†^			
≥18.2–24.9	6 (30)	2 (20)	4 (40)
≥25–29.9	6 (30)	3 (30)	3 (30)
≥30.0	8 (40)	5 (50)	3 (30)
Education ^†^			
<12th grade	4 (20)	1 (10)	3 (30)
12th grade	6 (30)	2 (20)	4 (40)
>12th grade	10 (50)	7 (70)	3 (30)
Marital status ^†^			
Married	17 (84)	10 (100)	7 (70)
Single	3 (16)	0 (0)	3 (30)
Language ^†^			
Spanish	15 (75)	7 (70)	8 (80)
English	5 (25)	3 (30)	2 (20)
Occupation ^†^			
Full time	8 (40)	5 (50)	3 (30)
Part time	5 (25)	2 (20)	3 (30)
House spouse	7 (35)	3 (30)	4 (40)

Values: * means (SD) or ^†^ number (%).

**Table 2:** Effects of DGA and Mexican adaptation interventions on serum biomarkers after 3 months.

Biomarker	Standard DGA			Mexican Adaptation			*p* ^0^	*p* ^1^
	Baseline	Follow-Up	Mean Difference	Baseline	Follow-Up	Mean Difference		
Cholesterol (mg/dL)	203.78 (177.8, 233.56)	192.88 (171.67, 216.70)	10.9	171.49 (155.96, 188.56)	164.06 (144.91, 185.73)	7.43	0.4	0.09
HDL (mg/dL)	57.83 (48.9, 68.4)	56.04 (46.51, 67.52)	1.79	56.41 (49.78, 63.92)	53.74 (46.31, 62.35)	2.67	0.73	0.62
LDL (mg/dL)	116.78 (96.27, 141.67)	108.85 (90.62, 130.74)	7.93	93.83 (79.46, 110.8)	86.97 (68.63, 110.21)	6.86	0.64	0.38
VLDL (mg/dL)	23.25 (15.03, 35.99)	20.30 (12.69, 32.49)	2.95	18.2 (13.64, 24.27)	18.95 (14.25, 25.18)	−0.75	0.49	0.25
Triglycerides (mg/dL)	115.48 (74.38, 179.31)	101.80 (63.46, 163.30)	13.68	90.96 (68.04, 121.61)	94.35 (70.97, 125.44)	−3.39	0.55	0.3
FFAs (mEq/L)	0.50 (0.41, 0.62)	0.37 (0.28, 0.47)	0.13	0.41 (0.33, 0.50)	0.45 (0.38, 0.52)	−0.04	<0.01	<0.01
Glucose (mg/dL)	103.43 (89.52, 119.5)	104.47 (89.80, 121.53)	−1.04	95.09 (82.83, 109.15)	97.22 (80.54, 117.36)	−2.13	0.43	0.37
Insulin (µU/mL)	7.79 (4.93, 12.32)	8.10 (5.43, 12.09)	−0.31	8.10 (5.4, 12.16)	7.98 (4.86, 13.09)	0.12	0.9	0.85
CRP (mg/dL)	0.25 (0.10, 0.60)	0.18 (0.06, 0.53)	0.07	0.19 (0.09, 0.38)	0.14 (0.07, 0.29)	0.05	0.92	0.74
HOMA-IR	1.99 (1.11, 3.55)	2.09 (1.24, 3.52)	−0.1	1.90 (1.14, 3.17)	1.91 (1.02, 3.59)	−0.01	0.54	0.55

Values are geometric means (95% CI). *n* = 10 for each arm. *p*^0^ is the *p*-value obtained from a GEE model comparing the changes in the biomarker from baseline to follow-up between the intervention and control groups—unadjusted. *p*^1^ is the *p*-value obtained from a GEE model comparing the changes in the biomarker from baseline to follow-up between the intervention and control groups—adjusted for age and BMI.

**Table 3:** Intervention effect on diet composition for 18 participants at 3 months.

Nutrient	Standard DGA ^a^			Mexican Adaptation ^b^			*p* ^1^
	Value at Baseline	3-Month Follow-Up	Mean Difference	Value at Baseline	3-Month Follow-Up	Mean Difference	
Energy (kcal/d)	1679 (1375, 2051)	1160 (899, 1498)	519	1496 (1104, 2027)	1434 (1092, 1884)	62	0.05
Carbohydrates (%E/d)	49.3% (44.7, 54.3)	49.4% (45.7, 53.4)	−0.1	49.8% (45.1, 54.9)	51% (47, 55.2)	−1.2	
Carbohydrates (g/d)	206.9 (161.8, 264.6)	143.3 (109.7, 187.3)	63.6	186.2 (135.8, 255.2)	182.7 (142.3, 234.7)	3.5	0.03
Total fat (%E/d)	34% (29.2, 39.7)	33.1% (28.3, 38.6)	0.9	32.9% (29.4, 36.8)	33.4% (30.1, 37.1)	−0.5	
Total fat (g/d)	63.5 (52.1, 77.4)	42.6 (30.8, 58.9)	20.9	54.7 (38.5, 77.8)	53.3 (38.2, 74.4)	1.4	0.09
Saturated fat (g/d)	19.9 (16, 24.8)	12.5 (9, 17.4)	7.4	17.2 (11.4, 26)	16.4 (12, 22.3)	0.8	0.12
Protein (%E/d)	16.7% (15.6, 17.9)	18.5% (16.2, 21.2)	−1.8	17.2% (15.6, 17.9)	14.5% (12.1, 17.3)	2.7	
Protein (g/d)	70.2 (57.3, 85.9)	53.7 (44, 65.7)	16.5	64.6 (48.1, 86.8)	51.8 (38.2, 70.3)	12.8	0.32
Dietary fiber (g/d)	21.2 (16.7, 26.9)	14.5 (9.9, 21.2)	6.7	20 (14.6, 27.2)	19.5 (14.5, 26.3)	0.5	0.08
Fruit (servings/d)	1.7 (1.1, 2.6)	1.7 (1, 2.8)	0	1.7 (1.3, 2.3)	1.8 (1.1, 2.9)	−0.1	0.07
Vegetable (servings/d)	2.2 (1.3, 3.5)	2 (1.2, 3.4)	0.2	1.5 (0.9, 2.5)	2.4 (1.7, 3.5)	−0.9	0.11

Values are geometric means (95% CI) or percentages. ^a^: *n* = 8 participants; ^b^: *n* = 10 participants. *p*^1^ is the *p*-value obtained from a GEE model comparing the changes in the dietary components from baseline to 3-month follow-up between the intervention and control groups—adjusted for age and BMI.

**Table 4:** Intervention effect on diet composition for 10 participants at 6 months.

Nutrient	Standard DGA ^a^					Mexican Adaptation ^b^					***p* ^1^**	***p* ^2^**
	Value at Baseline	3-Month Follow-Up	6-Month Follow-Up	Mean Difference 1	Mean Difference 2	Value at Baseline	3-Month Follow-Up	6-Month Follow-Up	Mean Difference 1	Mean Difference 2		
Energy (kcal/d)	1819 (1368, 2419)	1143 (804, 1624)	1194 (688, 2072)	676	625	1829 (1047, 3193)	1416 (868, 2310)	983 (618, 1562)	413	846	0.51	<0.01
Carbohydrate (%E/d)	49.8% (41.8, 59.3)	49.1% (45.6, 52.9)	52% (45.4, 59.5)	0.7	−2.2	49.1% (47, 51.3)	51.3% (49.5, 53.1)	48.8% (38.7, 61.7)	−2.2	0.3		
Carbohydrate (g/d)	226.5 (158.6, 323.5)	140.3 (101.6, 193.9)	155.1 (85.8, 280.5)	86.2	71.4	224.5 (125.6, 401.5)	181.5 (111.1, 296.6)	119.9 (69.6, 206.6)	43	104.6	0.39	0.02
Total fat (%E/d)	32.5% (24.9, 42.4)	33.7% (28.4, 39.9)	30.4% (24.9, 37)	−1.2	2.1	35.5% (33.6, 37.5)	34.3% (30.3, 39)	36.4% (28.4, 46.8)	1.2	−0.9		
Total fat (g)/d	65.7 (46.5, 93)	42.8 (26.5, 69.3)	40.3 (23, 70.7)	22.9	25.4	72.2 (41.8, 124.6)	54 (32.2, 90.8)	39.8 (23.4, 67.6)	18.2	32.4	0.83	0.03
Saturated fat (g/d)	21 (14.5, 30.5)	12.8 (7.9, 20.8)	13.2 (7.4, 23.3)	8.2	7.8	23.1 (12.3, 43.6)	16.4 (9.3, 28.9)	11.8 (6.7, 20.7)	6.7	11.3	0.9	0.01
Protein (%E/d)	17.1% (15.3, 19.1)	18.2% (15.1, 22)	16.7% (14.2, 19.7)	−1.1	0.4	16.2% (15.3, 17.1)	12.6% (7.1, 22.2)	11.5% (10.5, 12.7)	3.6	4.7		
Protein (g/d)	77.9 (62.7, 96.7)	52.1 (41.8, 64.8)	55 (35, 86.5)	25.8	22.9	73.9 (43.1, 126.7)	44.4 (29.8, 66.2)	37.8 (24.5, 58.2)	29.5	36.1	0.48	<0.01
Dietary fiber (g/d)	24.1 (14.5, 33.3)	13.1 (8.5, 20.3)	15.7 (9.1, 27.3)	11	8.4	22.1 (10.1, 48)	20.8 (12.5, 34.4)	13.9 (8.6, 22.4)	1.3	8.2	0.25	0.29
Fruit (servings/d)	1.6 (0.8, 3.4)	1.5 (0.8, 2.9)	1.3 (0.5, 3.6)	0.1	0.3	2.1 (1.4, 3.3)	2.1 (0.4, 9.9)	1.6 (0.6, 4.1)	0	0.5	0.08	0.71
Vegetable (servings/d)	2.1 (0.9, 5.3)	1.7 (1, 3.1)	2.2 (0.9, 5.6)	0.4	−0.1	1.4 (0.3, 7.1)	2.2 (0.8, 5.7)	1.5 (0.5, 4.5)	−0.8	−0.1	0.32	0.49

Values are geometric means (95% CI) or percentages. ^a^: *n* = 6 participants; ^b^: *n* = 4 participants. *p*^1^ is the *p*-value obtained from a GEE model comparing the changes in the dietary components from baseline to 3-month follow-up between the intervention and control groups—adjusted for age and BMI. *p*^2^ is the *p*-value obtained from a GEE model comparing the changes in the biomarker from baseline to 6-month follow-up between the intervention and control groups—adjusted for age and BMI.

**Table 5:** End-of-study questionnaire.

Question	Type of Question
1. How easy or difficult was it to understand the eating guide? Please tell us any thoughts you have on this	Likert scale and open-ended question
2. How easy or difficult was it to use the eating guide? Please tell us any thoughts you have on this	Likert scale and open-ended question
3. Which were the most useful parts of the eating guide or other materials we gave you?	Open-ended question
4. What tips were the easiest to adapt to your everyday life?	Open-ended question
5. Did you have any difficulties adapting the eating guide to your everyday life?	Open-ended question
6. What new or useful information do you feel you learned?	Open-ended question
7. Can you tell us 2 or 3 changes you made in your eating over the past 3 months? (If you didn’t make any changes please write “no changes”)	Open-ended question
8. Are there any changes you would like to continue with?	Open-ended question
9. What type of study communications were most useful? Our texts, phone calls, or conversation during clinic visit? Please give us your comments so we can do it better in the future	Open-ended question
10. Any other comments?	Open-ended question

**Table 6:** Summary of codes and examples for the first theme “Factors contributing to the adherence of the intervention”.

Codes	Standard DGA Arm	Mexican Adaptation Arm
Ease of use	“It’s very simple, it gives you freedom to adapt the foods according to your needs […]”	“It wasn’t difficult since it’s the Mexican guide and it’s what I already consume the most […]”
Important or interesting topic	“This topic is very interesting to me and there’s a lot of very beneficial information concerning our eating habits and how to prepare and consume foods”	“This was a fun and engaging study, personally. It forged a connection back to my culture and history as a Latina.”
Perception of healthiness	“Seeing my plate with the portions and a variety of vegetables is very healthy.”	“It seems like a very important topic to me, and it helps us realize that small changes can help us make a big difference in preparing healthier meals without huge sacrifices.”
Variety	“All is perfect, like the variety”	Not reported
Intervention components (included nine codes: guide, recipes, study staff, calls, text, email, in-person guidance, all components, and others)	“How they explained how much you should eat of each group” “All of the information is useful, but for me visuals like the recipes are easier.”	“The guide is easy to understand, basic and to the point.” “All of the communications were useful (texts/phone calls/in-person conversations) I think 3–4 weeks was an appropriate time to be checking-in.”
Extrinsic facilitators: family values	“Continue as I am now because I know it’s healthy. For my children, cooking at home is important.”	“I eat a lot of traditional Mexican food already, especially with my family”

**Table 7:** Summary of codes and examples for the second theme: “Factors negatively affecting adherence to the intervention”.

**Codes**	**Standard DGA Arm**	**Mexican Adaptation Arm**
Intrinsic barrier: lack of time	“Yes, my lack of time is always getting in the way. Exercise is difficult for me, finding the time.”	Not reported
Intrinsic barrier: challenge with specific dietary components	“I feel that dairy shouldn’t be pushed so much.”	“It was difficult to stop using lard, and with the recipes, the portions were difficult. The salt, I consume more salt. I know salt is bad, my doctor told me”
Other barriers (not included in the previous two codes)	“I liked the recipes, but I don’t always have the ingredients at home.”	“The guide was easy to understand, although I only make/eat traditional Mexican foods like pozole, menudo, etc. on special occasions.”
Extrinsic barrier: family pushback	“Yes, I had problems [in the form of] family pushing me to eat more meals at restaurants”	“Using more vegetables when cooking because I already eat vegetables. It’s difficult because my husband is more of a meat eater.”
Untimeliness of study materials	“Everything was good, but I would have liked to have received the recipes earlier at the beginning”	“Only complaint would be that it seemed we received some of the information too late, would have liked to have all materials at the beginning of the study.”

**Table 8:** Summary of codes and examples for the third theme: “Perceptions of specific dietary components”.

Codes	Standard DGA Arm	Mexican Adaptation Arm
Fruits and vegetables	“I realized I need to eat more veggies & fruits”	“Eating healthy, like fruits and vegetables”
Fats	“Avoiding red meats and fats (pork, beef)”	“Cooking with less fat because it is easier”
Sugar	“Cutting down on sugars, being more on top of my family’s sugar consumption”	“[…] tried to cut down on sugars, but it was a challenge”
Sugar-sweetened beverages	“No more soda, specifically Coca-Cola because of its sugar and caffeine content”	“Yes, switching from Coca-Cola to natural water”
Junk food/processed food (general)	“Tried to eat less junk food, smaller (decent) portions”	“No, just being more conscious to cut out more processed foods like potato chips, pizzas, fast food and cook more Mexican meals/dishes, which is the norm for me and my family anyway”
Water	Not mentioned	“Eating smaller portions and eating more fruits and vegetables and most of all, to drink a lot of water”
Salt/sodium	“Yes, follow a healthy eating pattern for my entire life and limit the consumption of sugars and saturated fats and sodium and also limit calorie consumption”	“The salt, I consume more salt. I know salt is bad, my doctor told me”
Meat	“That my stomach struggles to digest pork and beef”	“I got more ideas for how to cook meats and vegetables in meals”
Beans	“I’m eating a lot more beans than before, in the past I thought they made my [sic] gasy, but now I crave them. I used to burp a lot, now I don’t, not these past months.”	“Beans, they’re so good and dynamic, tostadas are a great vehicle for many other ingredients”
Flour	“Not eating white flour, I replaced it with whole wheat flour, or I avoided it”	“Reducing my flour [carb] intake and consuming more grains”
Others	“I tried to drink more pressed green juices, nothing added, just things like coconut water, turmeric.”	“I included more vegetables, nuts and eggs in my daily diet.”

**Table 9:** Summary of codes and examples for the fourth theme: “Information or practices learned from the intervention and suggestions”.

Codes	Standard DGA Arm	Mexican Adaptation Arm
Useful information learned from the intervention	“How to put together meals or dishes and how to economize and buying foods with only $10, fresh or frozen, and the amount that it can yield.”	“Knowing that making small changes and eating healthier can prevent chronic illnesses”
Portion sizes	“Reading the quantities of each portion, like granola bars, the amount of sugar it has, fat, etc.”	“Thankful for teaching us to eat smaller portions”
Frequency	Not reported	“Eating pattern recommendations and keeping in mind to try to eat 1–2 servings of beans each day, vegetables, corn based (masa), fruits, and whole milk.”
Changes made during the intervention	“The change I made was to have protein for breakfast every day and to carry healthy snacks/or have them at hand”	“1. More salsa = more vitamin C & other nutrients 2. Meal planning and cooking more”
Continuation of changes post-intervention	“Eating less processed flour products, using less dressing”	“Continue incorporating vegetables to the meals I prepare”
Exercise	“Complimenting with exercise”	“With exercise, and to include more fruits and vegetables in my diet”
Suggestions	“Something that would help busy people like me eat better, guides, food recommendations”	“The recipes could have been more useful if handed out towards the beginning! I’m sad I didn’t get to use them. More emphasis on things we should eat less of. Would’ve liked seeing an on-line (log-in required) sort of method for completing food questionnaires, like a virtual food diary.”

## Data Availability

The datasets and codes generated and/or analyzed during the current study are not publicly available as data sharing of this nature was outside the scope of the original study. However, the data may be available from the corresponding author if a data use agreement is submitted by the requester. All analyses were carried out using R statistical software version 4.0.2. The code is not publicly available; however, once the data agreement by the requester is completed, it could become available.

## Abbreviations

The following abbreviations are used in this manuscript:

DGA	Dietary Guidelines for Americans
BMI	Body mass index
GEE	General estimating equation
EAR	Estimated average requirement
FFQ	Food Frequency Questionnaire
NDSR	Nutrition Data System for Research
hsCRP	High-sensitivity *C*-reactive protein
HDL	High-density lipoprotein
LDL	Low-density lipoprotein
VLDL	Very low-density lipoprotein
HOMA	Homeostatic Model Assessment
FFAs	Free fatty acids
CV	Coefficient of variation
SNAP	Supplemental Nutrition Assistance Program
